# Mycobacterial Evolution Intersects With Host Tolerance

**DOI:** 10.3389/fimmu.2019.00528

**Published:** 2019-03-22

**Authors:** Joseph W. Saelens, Gopinath Viswanathan, David M. Tobin

**Affiliations:** ^1^Department of Molecular Genetics and Microbiology, Duke University School of Medicine, Durham, NC, United States; ^2^Department of Immunology, Duke University School of Medicine, Durham, NC, United States

**Keywords:** *Mycobacterium tuberculosis*, evolution, host tolerance, clinical phenotypes, mycobacteria, mycobacterial genomes

## Abstract

Over the past 200 years, tuberculosis (TB) has caused more deaths than any other infectious disease, likely infecting more people than it has at any other time in human history. *Mycobacterium tuberculosis* (*Mtb*), the etiologic agent of TB, is an obligate human pathogen that has evolved through the millennia to become an archetypal human-adapted pathogen. This review focuses on the evolutionary framework by which *Mtb* emerged as a specialized human pathogen and applies this perspective to the emergence of specific lineages that drive global TB burden. We consider how evolutionary pressures, including transmission dynamics, host tolerance, and human population patterns, may have shaped the evolution of diverse mycobacterial genomes.

## Introduction

Tuberculosis (TB) is a critical health crisis in our modern world. TB is one of the top ten causes of death worldwide, killing an estimated 1.7 million people in 2017 (1). Despite years of coordinated global efforts to reduce the burden of TB, it is estimated that around 10 million new infections developed around the world in 2017 (1).

*Mycobacterium tuberculosis* (*Mtb*), the etiologic agent of TB, has evolved through the millennia to become a highly specialized obligate human pathogen. Indeed, some consider *Mtb* as the archetypal human-adapted pathogen (2). Unlike the non-pathogenic soil-dwellers and the opportunistically pathogenic species of mycobacteria, *Mtb* has no known environmental reservoir and does not survive outside of its human host. For its survival, *Mtb* has evolved to subvert and co-opt the very mechanisms the human immune system deploys to clear bacterial infections for its own advantage. However, the host is capable of limiting mycobacterial growth and, in some cases, inducing latency (3, 4), or sterilizing the infection (5, 6). Latent or subclinical disease provides mechanisms whereby *Mtb* can remain in the host and reactivate following immune suppression, transmitting to new hosts (7), although our previous understanding of the nature and significance of latent disease is now being rethought (8, 9). Nonetheless, this balance between host and pathogen is central to the evolutionary survival strategy of *Mtb* as an obligate human pathogen. Indeed, it is estimated that 90% of people that are infected by *Mtb* either contain or clear the infection (10). Yet the 10% of patients who develop active disease transmit *Mtb* to such a degree that one quarter of the world's population is estimated to have mounted an immune response to the pathogen (11). TB has caused over 1 billion deaths in the past 200 years, surpassing all other infectious diseases (12). In this review, we discuss the features of *Mtb* that were central to its emergence as a human pathogen and how genetic diversity among strains contributes to phenotypic diversity in disease presentation, with a focus on the evolutionary interplay between pathogen and host. Bacterial factors that engage the host promote bacterial growth, survival, and transmission in human populations. Yet, overall, an evolutionary balance has been reached in which host mechanisms of containment and tolerance counteract many of these bacterial features.

### The Origins of *Mtb*

The timing of events that contributed to *Mtb*'s specialized adaptation to human hosts remains a matter of debate. Some point to an early origin of *Mtb* ~70,000 years ago (13, 14), while others have more conservative estimates of 35,000 years ago (15). Other studies suggest a more recent emergence of ~6,000 years ago is most likely (16). These estimates are based on different assumptions and study materials, and have therefore led to a wide dispersion of estimates.

**Figure 1:**
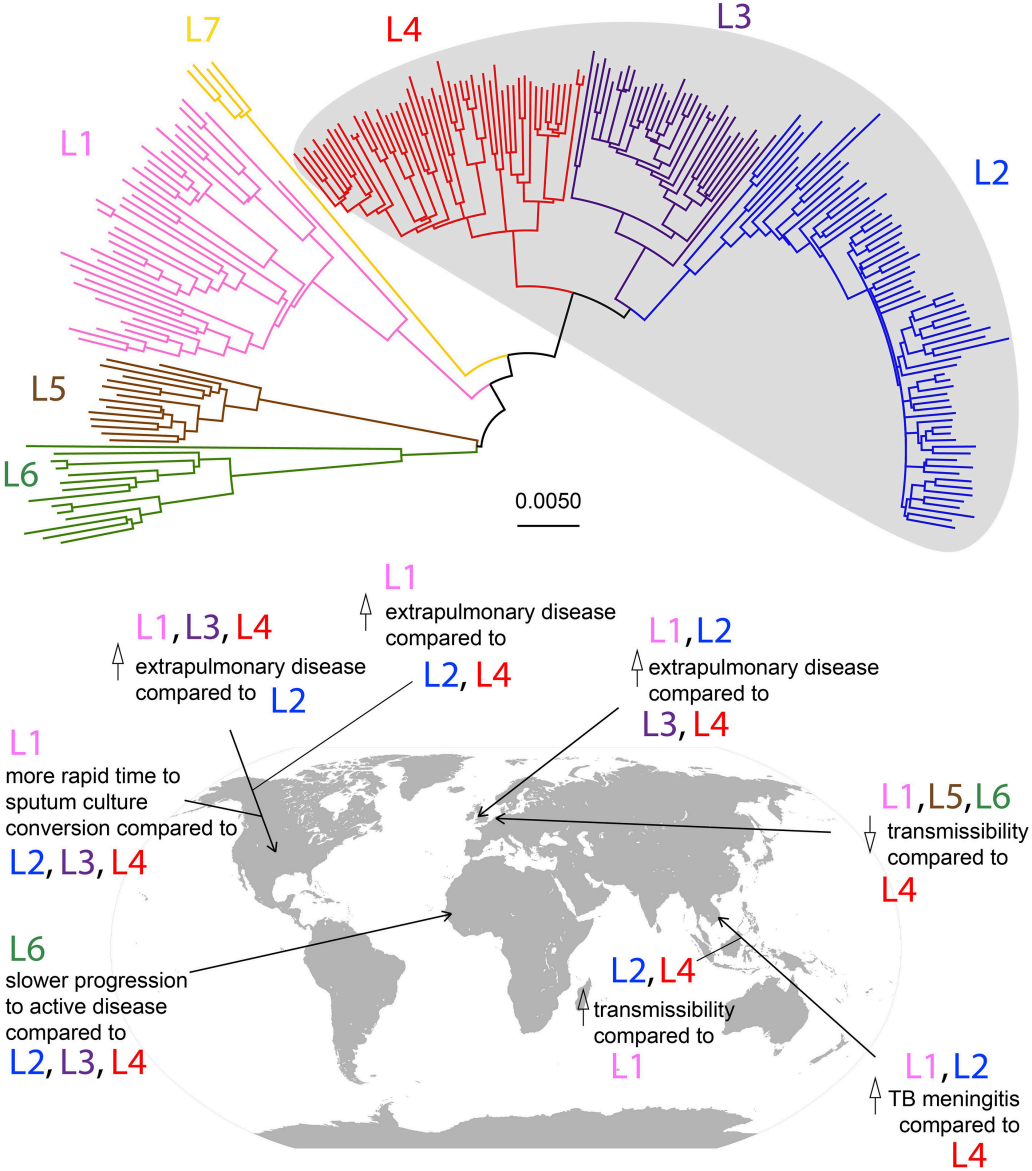
Phylogeny of *Mtb* lineages and geographic associations of disease characteristics. Neighbor-joining phylogeny based on 35,787 SNPs among 225 strains from Comas et al. (13). Lineages are color-coded according to the scheme described in Bos et al. (16), and modern lineages are shaded in gray. Scale bar represents relative number of substitutions per known variant. Disease characteristic associations with *Mtb* lineages in geographic locations by studies described in Table 1 are marked on a world map.

Most studies have employed inference methods based on DNA sequence among extant strains of *Mtb*. This method relies on the calibration of a molecular clock, which uses genetic distance as a measure of time since divergence (17). *Mtb* demonstrates a clonal population structure that can be divided into seven major lineages (Figure 1), and the divergence between these lineages and the other members of the *Mycobacterium tuberculosis* complex (MTBC) has been used by some to calibrate the molecular clock for *Mtb* (25).

Prior to the advent of widespread accessibility to whole genome sequencing, *Mtb*'s molecular clock was estimated using variable numbers of tandem-repeats (VNTR) in microsatellite-like loci (26). This method proposed an origin of the MTBC approximately 40,000 years ago, and highlighted the likelihood of *Mtb* dispersing throughout Africa and Eurasia via human migration (27). However, the use of VNTR in constructing phylogenies can lead to phylogenetic arrangements incongruent with known genetic relationships due to convergent evolution at these loci (28). Therefore, the current gold standard for calibrating a molecular clock is genome sequencing. However, as demonstrated below, the method by which *Mtb's* molecular clock is calibrated will have a significant impact on the resulting estimates.

Multiple studies have employed genome sequencing to determine the molecular clock of *Mtb* and have arrived at vastly different estimates for the age of *Mtb*. The calibration of the molecular clock underlies these differences. Comas et al. estimate *Mtb*'s origins as far back as 70,000 years ago (13). This estimate is based on the parallels of mitochondrial DNA (mtDNA) haplogroups and the lineages of *Mtb* that are most commonly found among the corresponding human populations, and then calibrating the molecular clock using key events in human evolution reflected by mtDNA. This generated an estimated mutation rate in *Mtb* of 2.58 × 10^−9^ substitutions/site/year, which is low compared to estimates derived from contemporary outbreaks (1.1 × 10^−7^ substitutions/site/year) (29). However, their estimates produced multiple time points for *Mtb*'s emergence, and 70,000 years was chosen as the most likely. The researchers who put forth this hypothesis on the origin of *Mtb* had previously published work proposing the dispersal of *Mtb* via human migration out of Africa (14). While the phylogeographic distribution of the major lineages of *Mtb* coincide with concordant patterns in human migration (25), calibrating *Mtb's* molecular clock based on these patterns to determine when *Mtb* emerged presupposes its own hypothesis that *Mtb* emerged with modern humans.

Others have challenged this hypothesis and proposed a much later time frame for *Mtb*'s emergence (30). Instead of mtDNA, Pepperell et al. based their estimates on historical samples of MTBC strains and determined that the emergence of the most basal species of *Mtb, M. africanum*, occurred approximately 2,200 years ago. The most recently evolved strains of *Mtb*, those among the so-called “modern” lineages, are estimated to have arisen ~1,300 years ago. The estimated mutation rate of *Mtb* from this study (1.3 × 10^−7^ substitutions/site/year) was significantly higher than that of Comas et al. Furthermore, based on this early estimate for the origin of *Mtb*, Pepperell et al. propose the estimates for human population divergence do not correlate with the divergence of the *Mtb* lineages, and therefore did not disperse concurrently (30). Another study has put forth an origin estimate similar to that of Pepperell et al. The mummified remains of human samples from Peru dated between AD 1028 and AD 1280 demonstrated skeletal lesions indicative of TB (31–33). Sequenced ancient DNA (aDNA) from these samples revealed disease was caused by *M. pinipedii*, a member of the *Mycobacterium tuberculosis* complex (MTBC) that primarily infects seals (16). Comparing the aDNA against a current strain of *M. pinipedii* generated an estimate of MTBC's emergence occurring 6,000 years ago, with a mutation rate intermediate to the estimates of Comas et al. and Pepperell et al. (4.6 × 10^−8^ substitutions/site/year). However, the reliance on aDNA comes with the caveat that post-mortem DNA decays due to physical and chemical damage, leading to strand breakage and the hydrolytic deamination of cytosine to uracil (34). Therefore, additional bioinformatic corrections must be implemented to sort out decay artifacts, leading to the possibility of erroneous or missed variant calls in aDNA samples.

The variety of conclusions from these studies demonstrates that the calibration of the molecular clock is critical to the resulting estimates, and raises the question as to how well-suited *Mtb* is for molecular clock estimations. The application of molecular clocks relies on satisfying certain assumptions that could be problematic when applied to *Mtb*: namely, a constant mutation rate through time and the broad applicability of this rate across lineages (17). It is not at all clear that the mutation rate of *Mtb* is stable over evolutionary time, as no study has been able to collect longitudinal data from historical samples. Additionally, the health status of human hosts across space and time is highly variable, creating different pressures on the infecting strains. Furthermore, even among the extant lineages of *Mtb*, which are much more closely related to each other than they are to other members of the MTBC, variable mutation rates have been observed (35). A recent analysis highlights the complexities, uncertainties, and limitations of different methods used to calibrate an *Mtb* molecular clock (36).

The earliest claim of mycobacterial disease comes from a 500,000 year old fossil of *Homo erectus*, which demonstrated lesions characteristic of mycobacterial infection (37). As no ancient DNA (aDNA) was recovered from this sample, it is impossible to determine what species of mycobacteria might have caused the lesions. Using lipid profiles unique to pathogenic mycobacteria and the *IS6110* insertion element (38), a feature found only in members of the MTBC (39), the oldest confirmed sample of mycobacterial disease was found in bovid fossils in North America, dating back approximately 17,000 years (40, 41). The earliest known association of the MTBC with humans comes from Atlit-Yam, Israel, dating back 9,000 years (42). Interestingly, this sample bears the TbD1 marker, a genomic deletion found exclusively in the evolutionarily “modern” lineages of *Mtb* (43). Linking definitive archaeological findings with aDNA sequencing will provide the most compelling evidence to settle the divergent estimates. As the techniques for collecting and sequencing aDNA continue to advance, our insight into *Mtb*'s origins will similarly improve, and we may better understand the evolutionary forces and constraints leading to modern *Mtb* and the nature of its interactions with its hosts.

### The Evolution of *Mycobacterium tuberculosis* as a Specialized Pathogen

Mycobacteria range from environmental, non-pathogenic species, to opportunistic pathogens that infect immune-compromised hosts, to professional pathogens. The vast majority of *Mycobacteria* are non-pathogenic in nature. Comparative genomic studies have revealed the evolutionary trajectory to pathogenicity, in which environmental mycobacteria acquired virulence loci and became opportunists, and opportunists adapted to their host environments to become professional pathogens. The pathogenic species include but are not limited to: *Mycobacterium ulcerans* (the agent of Buruli ulcer), *Mycobacterium leprae* (leprosy), *Mycobacterium marinum, Mycobacterium canetti*, and the range of species that make up the MTBC. The MTBC contains the closely related species of pathogenic mycobacteria that, together, cause the vast majority of TB. Several of these species are animal-adapted strains that cause disease across a range of mammalian species. These include *Mycobacterium bovis* (infecting cows), *Mycobacterium caprae* (goats and sheep), *Mycobacterium pinipedii* (seals and sea lions), *Mycobacterium microti* (voles), and *Mycobacterium orygis* (oryxes) (44, 45)*. Mtb* and *Mycobacterium africanum* cause the majority of disease in humans. Among all of these pathogenic mycobacteria, *M. tuberculosis sensu stricto* has emerged as the most prevalent mycobacterial species and one of the most historically successful human pathogens. The key features and events that underlie the adaptation of mycobacteria into a specialized pathogen are discussed below and have also been highlighted in previous reviews [e.g., (2)].

### From the Environment to New Hosts

The soil-dwelling mycobacteria *Mycobacterium kansasii* is an environmental, opportunistic mycobacterial pathogen closely related to the MTBC. This genetic relationship provides insight into the late-stage events conferring *Mtb*'s specialized adaptation that allowed it to expand and persist as an obligate human pathogen. Unlike the nonpathogenic mycobacterial species, *M. kansasii* contains an array of virulence determinants for host adaptation. There are five ESX loci in *Mtb*, and all five are present in *M. kansasii* (46). Furthermore, *M. kansasii* has expanded its set of PE/PPE proteins and, in fact, encodes a greater number of PE/PPE proteins than *Mtb* and other members of the MTBC. Despite these similarities *M. kansasii* is only rarely found in patients, whereas *Mtb* infection in humans is prevalent (47, 48). Therefore, the ESX secretion systems and its effectors are not sufficient to explain the pathogenicity of *Mtb*. Given the shared virulence features of *M. kansasii* with *Mtb* but their vastly different impact on global health, what other features separate *Mtb* from *M. kansasii*?

The enhanced virulence of *Mtb* may have been the result of acquiring pathogenicity islands via horizontal gene transfer (HGT) (49–52). Comparative genomics reveals the presence of 55 genes in *Mtb* absent from *M. kansasii* (51). The majority of these genes contain an unusual GC content for mycobacteria and appear in clusters flanked by the vehicles that provide mechanisms for HGT (mycobacteriophage genes, transposons, and toxin-antitoxin systems). Notably, some of these HGT-acquired genes, encoding factors responsible for cell adhesion (53), arresting phagosome maturation (54, 55), the production of PGLs that function in oxidative stress resistance (56) and modulation of the host immune system (57) have been implicated in *Mtb*'s adaptation to survival within a host (55, 58).

Mycobacterial species comprising the “smooth tubercle bacilli” (STB) are thought to be an evolutionary bridge between the environmental opportunistic species, such as *M. kansasii*, to the pathogenic MTBC (46). Unlike the MTBC, genome sequencing reveals that *M. canetti* demonstrates a non-clonal population structure with >60,000 SNPs separating some strains (50). While the environmental reservoir of *M. canetti* remains unknown, cases are highly geographically restricted and arise predominantly in patients who have some form of contact with East-Africa (59). Like *M. kansasii, M. canetti* harbors compelling signatures of HGT in its genome (60, 61). Boritsch et al. offered conclusive experimental evidence that HGT occurs in *M. canetti*, finding the transfer of DNA fragments as large as 117.6 kilobase pairs (kbp) (62). Like *M. canetti*, the most basal lineages in the MTBC, including L5, L6, and L7, are also strongly geographically restricted to Africa (14, 63, 64). These observations and experiments support a scenario in which an *M. canetti*-like species of mycobacteria in Africa acquired virulence loci via HGT, thus giving rise to the pathogenic progenitor of the MTBC.

The role of ongoing HGT in *Mtb*, however, remains controversial. Most evidence suggests that *Mtb* demonstrates clonal evolution without ongoing recombination events. In the same experiments in which HGT was detected in *M. canetti*, HGT could not be detected among MTBC species (62). The lack of ongoing HGT in the MTBC is supported by the congruence of phylogenetic trees based on a variety of molecular markers (65–67), stable G+C content across the majority of the genome (68), a low frequency of homoplasic mutations (14, 28), and that all known drug-resistance factors arise via *de novo* mutation (69). The mechanism by which *Mtb* lost capacity for ongoing genetic recombination, however, remains unknown. Together, this evidence provides strong support for the role of HGT as a critical component in the emergence of *Mtb*, and that subsequently *Mtb* appears to have lost significant capacity for genetic recombination and evolved in a clonal fashion.

### Genetic and Phenotypic Diversity in *Mtb*

*Mtb* is an obligate human pathogen and has no known environmental reservoir. As such, its population structure is largely isomorphic to its human host population. Despite the clonal evolution of *Mtb*, significant genetic variation exists and based on this it is divided into seven major lineages. These lineages can be grouped into evolutionarily “ancient” and “modern” lineages, with the TbD1 deletion serving as a genetic marker separating the two groups (43). The ancient lineages (L1, L5, L6, L7) demonstrate a high degree of geographic constraint (14, 63, 64), whereas the more recently evolved modern lineages (L2, L3, L4) are found more broadly throughout the world (70). L1 predominantly circulates in Southeast Asia, L5 and L6 in West Africa, and L7 in the Horn of Africa. L2 is strongly associated with an East Asian origin (71), but also causes significant disease burden in Eurasia, South Africa, and Peru. Over the past 200 years, the population size of L2 strains has dramatically increased, and can be found in most countries throughout the world (72). L3 strains circulate mostly in India and Central Asia. L4 strains cause the most global disease and are the most widely distributed among the *Mtb* lineages (73). Interestingly, discrete sublineages within L4 differ in their geographic distribution, suggesting that some L4 strains are more capable of spreading to new host populations (74).

The genetic lineages of *Mtb* were first defined by lineage-specific deletions, referred to as large sequence polymorphisms (LSPs) (25). Due to the extreme rarity of ongoing horizontal gene transfer (HGT) among species of the MTBC, these markers are thought to be largely irreversible and well-suited to lineage classification (73). Single nucleotide polymorphisms (SNPs) are also phylogenetically informative in *Mtb* due to the lack of ongoing HGT, and help to increase the resolution of relationships among strains within a lineage (75–77). From the application of these markers in constructing the phylogenetic relationships among *Mtb* lineages, it has become clear that the ancestral lineages separate into distinct phylogenetic groups, and are thus paraphyletic in nature. The modern lineages, conversely, are more closely related and share a more recent common ancestor (i.e., monophyletic) than the ancient lineages are with one another. These lineages have evolved independently in separate human populations, resulting in distinct induction of inflammatory phenotypes (78, 79) and differential modulation of innate immune signaling (80). Furthermore, the variable geographic distribution and disease burden of the different lineages raises the question as to how the existing variation among *Mtb* strains contributes to disease phenotype, and whether this variation explains the uneven distribution of *Mtb'*s lineages.

### Phenotypic Diversity Among *Mtb* Lineages

Strain variation in disease severity, transmission potential, and resistance to drug therapy is of significant interest to global health. Identifying virulent and/or drug-resistant clones informs current and future treatment. Numerous studies have investigated the phenotypes associated with the different lineages and strains of *Mtb*. By the mid-20th century, TB research had begun to investigate virulence traits among clinical and reference strains of *Mtb* (81, 82). The first attempts to correlate virulence with strain background via typing techniques, however, did not occur until 1978 (83). In a landmark study, Valway et al. utilized *IS6110* typing patterns to identify a strain associated with a particularly virulent outbreak (84). The outbreak was characterized by extensive transmission among patients, and the researchers correlated a significant increase in *in vivo* replication as a potential underlying cause using a mouse infection model. Following the adoption of the restriction fragment length polymorphism (RFLP) typing technique (85) to describe the population structure of *Mtb*, strains originating in China and Mongolia, the so-called “Beijing” strains (now known as L2), demonstrated increased replication in cell culture and mouse models in addition to increased mortality *in vivo* (86, 87). In a rabbit infection model, L2 strains rapidly disseminated to extrapulmonary sites resulting in severe meningeal disease presentation (88). However, we should exercise caution when applying strain-specific characteristics broadly across its genetic lineage, as infection phenotypes can vary widely among strains from the same lineage (79, 89). Correspondingly, L2 strains demonstrate variable virulence patterns. The most recently evolved L2 strains, those comprising the so-called “modern Beijing” sublineage, exhibit increased virulence compared to the ancestral strains (90). These and earlier studies (91, 92) focused attention on the apparent increased virulence of the L2 strains, and their impact on the immune response was identified as an avenue of future research.

### *Mtb* Lineages and Disease Presentation

Transmission of *Mtb* depends on disease within pulmonary tissue in human hosts. Given its status as an obligate human pathogen, there are no environmental reservoirs for *Mtb* to transmit from, and extrapulmonary sites do not afford transmission. This leads to the question: Do particular *Mtb* lineages demonstrate variable disease presentations? Are more transmissible strains less often be associated with non-transmissible disease sites, i.e., extrapulmonary tissues? In a marmoset model of infection, a strain from the ancient L6 group was found to develop lower bacterial load in pulmonary tissue compared to modern strains from L2 and L4, but disseminated to extrapulmonary sites more compared to L4 (93). Interestingly, the L2 strain demonstrated the highest burden in all organs assayed, effectively replicating within the lung and disseminating to extrapulmonary sites. This study suggests the modern strains are more capable of transmitting by establishing pulmonary disease, but L2 also spreads effectively to extrapulmonary sites. Based on the characteristics of infection, it is possible that the L2 and L6 strains disseminated to other tissues by different mechanisms, where L2's dissemination was a byproduct of increased overall virulence as described in the preceding sections. While this study offers novel visualization methods to assess disease progression of diverse tuberculosis lineages in a primate infection model, it is not clear how generalizable these phenotypes are across these lineages.

There are few studies that have compared patterns of disease presentation among a diverse range of strains from more than two lineages in a large sample population (summarized in Figure 1 and Table 1). Even in these, associations between lineage and disease presentation have been variable, and the comparisons differ. In the United States, L1, L3, and L4 strains were more likely to cause extrapulmonary disease compared to strains from L2 (20). In Vietnam, L1 and L2 strains were associated with TB meningitis compared to L4 strains (18). In the UK, L1 and L2 were associated with increased likelihood of exclusively extrapulmonary disease compared to L3 and L4 (22). Aside from site of disease, characteristics such as time to sputum culture conversion and transmissibility differ between lineages as well. In the United States, L1 strains demonstrate a more rapid time to sputum culture conversion compared to strains from the modern lineages (L2, L3, and L4) (21). Additionally, in Gambia, L6 strains progressed to active disease at a significantly lower rate compared to strains from the modern lineages, but displayed no differences in transmissibility (19). However, in the Netherlands, ancient strains (L1, L5, and L6) demonstrated reduced transmissibility compared to L4 strains (23). In Florida, L1 strains were associated with higher rates of extrapulmonary disease compared to L2 and L4 strains (24). Together, these studies indicate that significant differences exist in disease presentation among the different lineages of *Mtb* (particularly between ancient and modern strains), and these patterns can be observed experimentally and in human populations.

**Table 1:** Studies investigating multiple *Mtb* lineages and their associations with disease characteristics.

**Geographic location**	**Lineages under study**	**Strain typing method**	**Lineage associations**	**References**
Vietnam	L1, L2, L4	IS*6110* RFLP, spoligotyping, MIRU-VNTR, & LSP	L1 and L2 cases higher odds of TB meningitis compared to L4	(18)
Gambia	L2, L4, L6	LSP	L6 infections less likely to progress to active disease compared to L2 and L4	(19)
USA	L1, L2, L3, L4	Spoligotyping & MIRU-VNTR	L1, L3, L4 cases higher odds of extrapulmonary tuberculosis compared to L2	(20)
USA	L1, L2, L3, L4	Spoligotyping & MIRU-VNTR	L1 more rapid time to positive sputum culture conversion	(21)
United Kingdom	L1, L2, L3, L4	MIRU-VNTR	L1 and L2 increased likelihood of exclusively extrapulmonary disease compared to L3 and L4	(22)
Netherlands	L1, L2, L3, L4, L5, L6	RFLP and MIRU-VNTR	L1, L5/L6 reduced transmission compared to L4	(23)
USA	L1, L2, L3, L4	Spoligotyping & MIRU-VNTR	L1 higher odds of extrapulmonary disease compared to L2 and L4	(24)

*RFLP, Restriction Fragment Length Polymorphism; MIRU-VNTR, Mycobacterial Interspersed Repetitive Unit-Variable Number Tandem Repeat; LSP, Large Sequence Polymorphism*.

### Bacterial Determinants of Virulence

*Mtb* lineages show varying geographic distribution patterns with ancient lineages being geographically restricted in comparison to the modern lineages. Several factors like population density, migration pattern, economic and health conditions, and more recently the HIV/AIDS pandemic and emergence of MDR strains could influence this distribution (94). However, bacterial genetic variation within and between each lineage may reflect evolutionary history and pressures.

### Cell Envelope-Associated Lipids

**Figure 2:**
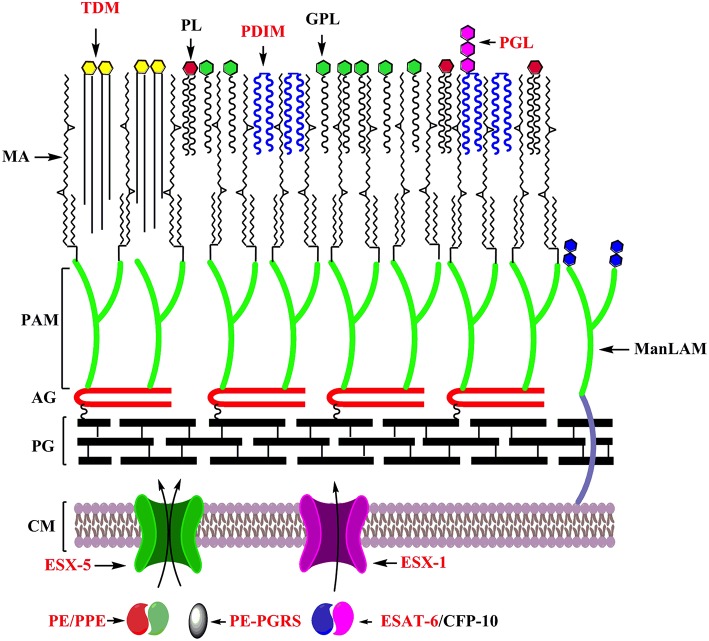
Key features underlying the adaptation of mycobacteria as specialized pathogens: Cell envelope of mycobacteria with factors playing distinct roles in its adaptation as a specialized pathogens labeled in red. CM, Cell membrane; PG, Peptidoglycan; AG, Arabinogalactan; PAM, Penta arabinosyl motif; MA, Mycolic acids; TDM, Trehalose dimycolate; PL, Phospholipids; PDIM, Pthiocerol dimycocerosate; GPL, Glycopeptidolipids; PGL, Phenolic glycolipids; ManLAM, Mannose capped lipoarabinomannan.

As a pathogen, *Mtb* must interface with its host, and the mycobacterial cell envelope makes first contact. The mycobacterial cell envelope is a complex multi-layered structure containing the plasma membrane, cell wall skeleton, mycomembrane and a capsule (95–97). It contains several lipids unique to pathogenic mycobacteria which contributes to their *in vivo* survival by modulating the host immune response, and have been the subject of more comprehensive reviews [e.g. (98)]. These include mannose capped lipoarabinomannan (ManLAM), phenolic glycolipid (PGL) and phthiocerol dimycocerosate (PDIM) (99–101). These features are highlighted in Figure 2.

Variations in these components among different strains and lineages may correspond to discrete evolutionary trajectories. For example, variation in ManLAM has been observed in clinical strains leading to altered virulence (102, 103). A subset of lineage 2 strains with truncated and more branched forms of ManLAM exhibited defects in phagocytosis by primary human macrophages when compared to lineage 4 reference strains (103).

Variations in PDIM, PGL and other lipids may also contribute to disease progression. PDIM can neutralize oxidative and nitrosative free radicals and has been proposed to play a role in protecting *Mtb* from these stress causing agents (104, 105). Further, PDIM may also have a role in immune evasion by masking cell wall pathogen-associated molecular patterns (PAMPs) (57), and also is required for proper secretion of ESX-1 substrates (106). Among the modern lineages, L2 strains but not L4 strains produce the phenolic glycolipid PGL, which may play an important role in promoting their virulence and transmission (107). In mycobacterium-infected macrophages, PGL induces the production of chemokine CCL2 which recruits monocytes to the site of infection. This facilitates mycobacterial escape from bactericidal macrophages to permissive monocytes (108). A point mutation in *Rv2952* encoding the S-adenosylmethionine-dependent methyltransferase in Beijing strains resulted in structural variations in PDIM and PGL compared to other lineage strains (109). As noted above, a deletion in the *pks1/15* locus encoding a polyketide synthase in L4 strains leads to defective production of PGL (110). These lipids can also inhibit the production or secretion of proinflammatory cytokines by the host leading to the establishment of infection (105, 107, 111, 112).

The abundant cell wall lipid trehalose dimycolate (TDM) plays multiple roles in pathogenesis (113–118). Specific cyclopropane modifications to the mycolic acids that comprise TDM are associated with pathogenic mycobacteria, but not with non-pathogenic species; PcaA-mediated modification of TDM modulates the host immune response to mycobacterial infection (119, 120). This cyclopropanated TDM plays an important role in inducing or accelerating host angiogenesis around the mycobacterial granuloma, a response that helps to support bacterial growth during early infection (121–123). Thus, intricately structured and complex lipid species provide important host modulatory activities and may be important substrates for evolution. Notably, lineage-specific differences in cytokine induction upon exposure of macrophages to lipid extracts from different lineages have been reported (78).

### Type VII Secretion Systems

The ESAT-6 secretion (ESX/Type VII) systems and their secretion substrates are key features that contribute to the pathogenicity of *Mtb* (124). The ESX secretion systems were discovered after genomic analysis of the *M. bovis* BCG vaccine strain revealed a large deletion [Region of Difference (RD) 1] that interrupted the ESX-1 system (125). This system was lost in *M. bovis* following 11-year serial culture by Calmette and Guerin in the pursuit of a TB vaccine. The absence of this system was subsequently shown to account for a significant share of BCG's attenuation, and much attention has been paid to the role of this and other ESX systems and their secreted substrates on *Mtb*'s virulence (126, 127).

ESX secretion systems are encoded in clusters throughout mycobacterial genomes. *Mtb* contains five ESX loci, which have expanded through gene duplication, diversification, and insertions of the ancestral ESX-4 locus (128). These clusters share six core genes encoding: three ESX conserved-components (EccB, EccC, EccD), a mycosin (MycP), and two small, secreted Esx proteins. Besides the most ancestral ESX-4 locus, the ESX clusters also encode genes for PE, PPE, EccA, EccE, and ESX-1-specific component (Esp) proteins. The *esp* genes are not specific to ESX-1, but they are most abundant in that system. Orthologs of ESX-4 can be found among mycobacterial and non-mycobacterial species in the phylum Actinobacteria (128, 129). ESX-4 is the simplest gene cluster among the ESX secretion systems, containing only seven genes. ESX-4 encodes the FtsK/SpoIIIE protein EccC4, the WXG proteins EsxU and EsxT, the conserved ESX core components EccB4 and EccD4, the mycosin protease MycP4, and the hypothetical valine and alanine rich protein Rv3446c.

The components of the ESX systems can be divided into cytosolic, membrane bound, and secreted proteins. EspG and EccA function in the cytosol. EspG is found in all ESX clusters besides ESX-4, and is thought to function as a specific chaperone for PE and PPE proteins (130–132). EccA is an AAA+ family (ATPase associated with various cellular activities) protein that is thought to form a hexamer and functions in the secretion of Esx and PE-PPE proteins (133–136). The conserved membrane components of ESX secretion systems (EccB, EccC, EccD, EccE, and MycP) are essential for secretion in all of the studied loci (137–141). These proteins contain large hydrophilic domains in either the N- or C-terminus and a range of transmembrane domains. EccB, EccC, EccD, and EccE are thought to form the transport channel through which the ESX substrates are transported across the inner membrane. EccB, EccC, EccD, and EccE form a stable membrane complex of ~1,500 kDa that can be co-immunoprecipitated (139). MycP, a mycosin, is a subtilisin-like protease containing a C-terminal transmembrane domain that tethers the protein on the cell membrane (142, 143). Its role in secretion remains unknown.

The components described thus far have been localized to the inner membrane. The inner membrane, however, is surrounded by a thick, lipid-rich cell wall (also referred to as the outer membrane or mycomembrane) in addition to another thick capsular layer [reviewed in (144)]. How ESX substrates are exported beyond these structural boundaries has been a mystery. Recently, Lou et al. discovered that EspC forms a long filamentous structure that localizes to the cell membrane, and its expression is required for secretion of EsxA (145).

The conserved secreted effectors of ESX systems are comprised of Esx and PE/PPE proteins (the latter is described in more detail in the following section). The Esx proteins are also referred to as WxG100 proteins due to a conserved tryptophan-X-glycine motif that causes a turn between two helical domains in the ~100 amino acid proteins (146). The most well-studied Esx proteins are EsxA and EsxB, encoded within the ESX-1 locus. ESX-1, the prototypical ESX secretion system in tuberculosis research, has been demonstrated to be essential for the intracellular survival of *Mtb* due to its critical role in host-pathogen interaction during *Mtb* infection via secretion of its substrates, many of which are secreted in a codependent manner (147). EsxA and EsxB are secreted as antiparallel heterodimers (148, 149) via recognition of an ESX secretion signal on the C-terminus of EsxB (150). EsxA has long-been associated as a cytolytic virulence factor of *Mtb* (126, 135, 151, 152). Experiments demonstrating recombinant EsxA could induce its cytolytic effect in the absence of infection led to the notion that EsxA was primarily responsible for ESX-1's pathogenicity (152, 153). However, recent work has definitively demonstrated that the cytolytic effect of recombinant EsxA was due to a residual detergent in the extract (154). Therefore, the cytolytic effect is dependent on other factors dependent on *Mtb*'s ESX-1 secretion system.

ESX-1 has been ascribed numerous roles in *Mtb*'s pathogenesis. As previously mentioned, ESX-1 is required for membrane disruptions in its host cell, allowing *Mtb* to escape from the phagosome and enter the cytosol whereupon necrosis-like cell death is induced (155–157). While EsxA has been shown to be insufficient to induce membrane disruptions, this process is dependent on its presence and secretion (154). EspB, which is encoded outside of the ESX-1 locus and depends on secretion of EsxA and EsxB for its own secretion, forms a ring-shaped heptamer with a hydrophobic domain, suggesting the possibility that it could be involved in membrane disruption via EsxA and EsxB (158). EsxA has been shown to induce expression of matrix metalloproteinase-9 (MMP9), which recruits additional phagocytes to the site of infection and facilitates its spread to new cells (156). The recurrent recruitment of additional leukocytes to take up the apoptotic debris of the former round of infected macrophages amplifies the bacterial population in successive waves and leads to the formation of the tuberculosis granuloma (159).

The regulation of ESX-1 differs among MTBC species, perhaps contributing to distinct infection phenotypes among lineages. The PhoPR regulon, a two-component regulation system, regulates the production and secretion of, among other things, EsxA and EsxB (160), and is central to the pathogenesis of *Mtb* (161). Strains from L5, L6 and the animal-adapted species all contain a missense mutation in *phoR* that downregulates the PhoPR system when genetically transferred into L2 and L4 strains (162). Intriguingly, Gonzalo-Asensio et al. noted that there were no significant differences in the production of proteins induced by PhoPR in the L5, L6 and animal-adapted species compared to L2 and L4, and that a deletion found only in the former rescued the defect. The authors went on to show that an outbreak of an unusually virulent strain of *M. bovis* that was transmitting among humans was associated with the insertion of an *IS6110* sequence upstream of *phoP*, serving as a promoter to increase the expression of the PhoPR regulon (162).

The pathogenic species of mycobacteria possess two additional ESX secretion systems, ESX-2 and ESX-5, that are not found in the rapid-growing, non-pathogenic mycobacteria (128, 163). The duplication of these systems in pathogenic mycobacteria is linked to the expansion of the PE and PPE gene families (163). The PE and PPE proteins, the other core substrates of ESX secretion systems, and their role in pathogenesis are discussed below.

### PE/PPE Family Proteins

Initial sequencing of the *Mtb* genome led to a surprise finding that 10% of its genes code for a unique family of proteins with signature proline-glutamate and proline-proline-glutamate residues conserved at their N-termini, linked to a variable C-terminus. Due to their variable C-termini, initially they were thought to be a source of antigenic variation to evade host immune system (68). The *pe*/*ppe* genes have greatly expanded in the pathogenic species of mycobacteria and have been critical for host adaptation (164, 165). This family of proteins are thought to help in *Mtb* survival and dissemination through diverse modes. This includes upregulation of anti-inflammatory cytokine levels (166), induction of apoptosis in macrophages (167) and increased secretion of chemokine MCP-1 (168). They also interact with TLR-2, leading to macrophage activation, promote apoptosis and necrosis in host cells (164). PE-PGRS a subfamily of PE family is unique to MTBC and related species (165). Mutations in their corresponding genes have been associated with impaired replication and decreased persistence in the host indicating a direct role for this class of genes in virulence (169). The “modern” Beijing strains from L2 have been demonstrated to harbor a deletion affecting *ppe38*, a consequential mutation that increases the virulence of affected strains (170). The authors found that the absence of *ppe38* inhibits the secretion of a large number of PPE_PGRS and PPE_MPTR (major polymorphic tandem repeats) substrates through ESX-5, and postulate that this mutation played a significant role in the global spread of the “modern” Beijing L2 strains. Thus, variation in these gene classes may contribute to the degree of virulence, transmissibility, and evolutionary success for mycobacterial species and strains within discrete hosts and genetic backgrounds.

### Mycobacterial Genetic Diversity and Its Intersection With Host Tolerance

Variation in mycobacterial lipids, ESX secretion systems and their effectors among the genetic lineages and sublineages of *Mtb* intersect with the nature of the host response to mycobacterial infection. Evidence from experimental infection models suggests that different *Mtb* lineages exhibit diverse growth phenotypes and elicit variable host immune responses. Hence in addition to these factors, the role of variable host tolerance among these lineages in shaping their diversity may be important. Some of the first evidence supporting this argument came from aerosol infections in mice with *Mtb* strains CDC1551, HN878, and HN60. CDC1551 belongs to lineage 4 whereas HN878 and HN60 belong to lineage 2. Mice infected with HN878 and HN60 succumbed earlier. This observation correlated with the cytokine profiles of CDC1551 infected mice which showed increased production of pro inflammatory cytokines TNF-α, IL-12, and IFN-γ in comparison to HN878 and HN60 infected mice (91). Moreover, strains from the modern lineages 2, 3, and 4 induced significantly lower levels of pro inflammatory cytokines than ancient lineages in a human monocyte-derived macrophages infection model (79).

*Mtb* sublineages too exhibit significant differences in virulence and immune modulatory functions. The M—Strain, a highly prevalent strain in Argentina belonging to the Haarlem family of Lineage 4 failed to induce PMN apoptosis and ROS production as opposed to the LAM family of the same lineage (171). Collectively these findings may help explain the emergence and evolutionary success of the modern lineages.

Recent work on tolerance in animal models of TB suggests that specific host factors can contribute differentially to bacterial restriction and host tolerance. For example, Phox-deficient mice are not compromised for resistance to infection but do display tolerance defects (172–174). Similarly, previous work in the zebrafish model of mycobacterial infection suggested that, in addition to overall bacterial load, inflammatory state influences disease outcome (175, 176). Thus, the degree of host tolerance to infection has important consequences to host survival, bacterial burden, and presumably transmission; indeed the majority of humans who do not manifest active disease upon exposure to *Mtb* suggests a high level of tolerance to infection (177). Reciprocally, how variation within distinct bacterial lineages and strains influences inflammation, tolerance, pathogenesis, and ultimately successful transmission, may determine the evolutionary trajectories of both pathogen and host.

A number of examples exist in which bacterial-host interactions appear to be specific to lineage. For example Lineage 2 mediated TB has been shown to be associated with C allele of *TLR-2*—T597C, and *NRAMP1*—D543N polymorphisms (18, 178). The−261TT variant in the Immunity-related GTPase Family M (IRGM) confers defense against pathogens including Lineage 4 *Mtb* which lacks *pks1/15*, but is not associated with *M. africanum* mediated TB. This gene is associated with PGL biosynthesis highlighting a potential role of the lipid in inhibiting IRGM mediated autophagy (179). Lineage 4 contains both ubiquitous (presumed to be generalist) and specialized (geographically restricted) sublineages, suggesting that at least some *Mtb* strains may have specialized to specific host populations (74). More recently, a large study in a Vietnamese population identified increased transmission of Lineage 2 Beijing strains between individuals than endemic strains, consistent with previous studies of transmission of Beijing strains in other regions (180–182). These studies underscore the need for further research that integrates data on *Mtb* strains and lineages with human genotypes to understand how this intersection contributes to the clinical outcome of *Mtb* infection. Ongoing studies with larger cohorts and deeper descriptions of clinical phenotypes should provide additional insight into these interactions.

*Mtb* genetic diversity and evolution may reflect the genetic arms race between successful pathogen and its host, leading to reciprocal genetic changes. There is newfound appreciation that host tolerance to mycobacterial infection is an important component of this interplay, contributing to disease trajectory and transmission patterns. Thus, genetic variation in aspects of host tolerance—generated through both bacterial and host mechanisms—is another important consideration in understanding the complex interactions between host and pathogen that have evolved during the long association between *Mtb* and its human hosts.

## Author Contributions

All authors listed have made a substantial, direct and intellectual contribution to the work, and approved it for publication.

### Conflict of Interest Statement

The authors declare that the research was conducted in the absence of any commercial or financial relationships that could be construed as a potential conflict of interest.
